# Single-Cell Time-Lapse Observation Reveals Cell Shrinkage upon Cell Death in Batch Culture of Saccharomyces cerevisiae

**DOI:** 10.1128/mBio.03094-21

**Published:** 2021-12-21

**Authors:** Setsu Kato, Kenta Suzuki, Taiki Kenjo, Junya Kato, Yoshiteru Aoi, Yutaka Nakashimada

**Affiliations:** a Graduate School of Advanced Sciences of Matter, Hiroshima University, Higashi-Hiroshima, Hiroshima, Japan; b Graduate School of Integrated Sciences for Life, Hiroshima University, Higashi-Hiroshima, Hiroshima, Japan; c School of Engineering, Hiroshima University, Higashi-Hiroshima, Hiroshima, Japan; Harvard Medical School

**Keywords:** budding yeast, cell death, stationary phase

## Abstract

Saccharomyces cerevisiae is a model organism for aging and longevity studies. In a clonal population of S. cerevisiae, the timing of cell death in the stationary phase is not synchronized, indicating that heterogeneity exists in survival at a single-cell level. Heterogeneity also exists in the cell size, and its correlation with the death rate has been discussed in past studies. However, the direct cause of the heterogeneity in survival remains unknown. In this report, we revisited this question and asked whether the death rate has any correlation with cell size. Past studies did not exclude a possibility that cells change their size upon or after death. If such a change exists, the size dependence of cell death could be misinterpreted. Therefore, we analyzed the correlation between the death rate and cell size before death by time-lapse imaging. It turned out that the size dependence of the death rate varied from one strain to another, suggesting that general principles between cell size and death do not exist. Instead, cells shrink upon cell death, resulting in the accumulation of small dead cells. The degree of cell shrinkage was proportional to the cell size, and the ratio was constant in two strains, which is between 25 and 28%, suggesting the presence of general principles and mechanisms behind the shrinkage event upon cell death. Further investigation of the cause and mechanism of the shrinkage will help us to understand the process of cell death and the origin of the heterogeneity in survival.

## OBSERVATION

**FIG 1:**
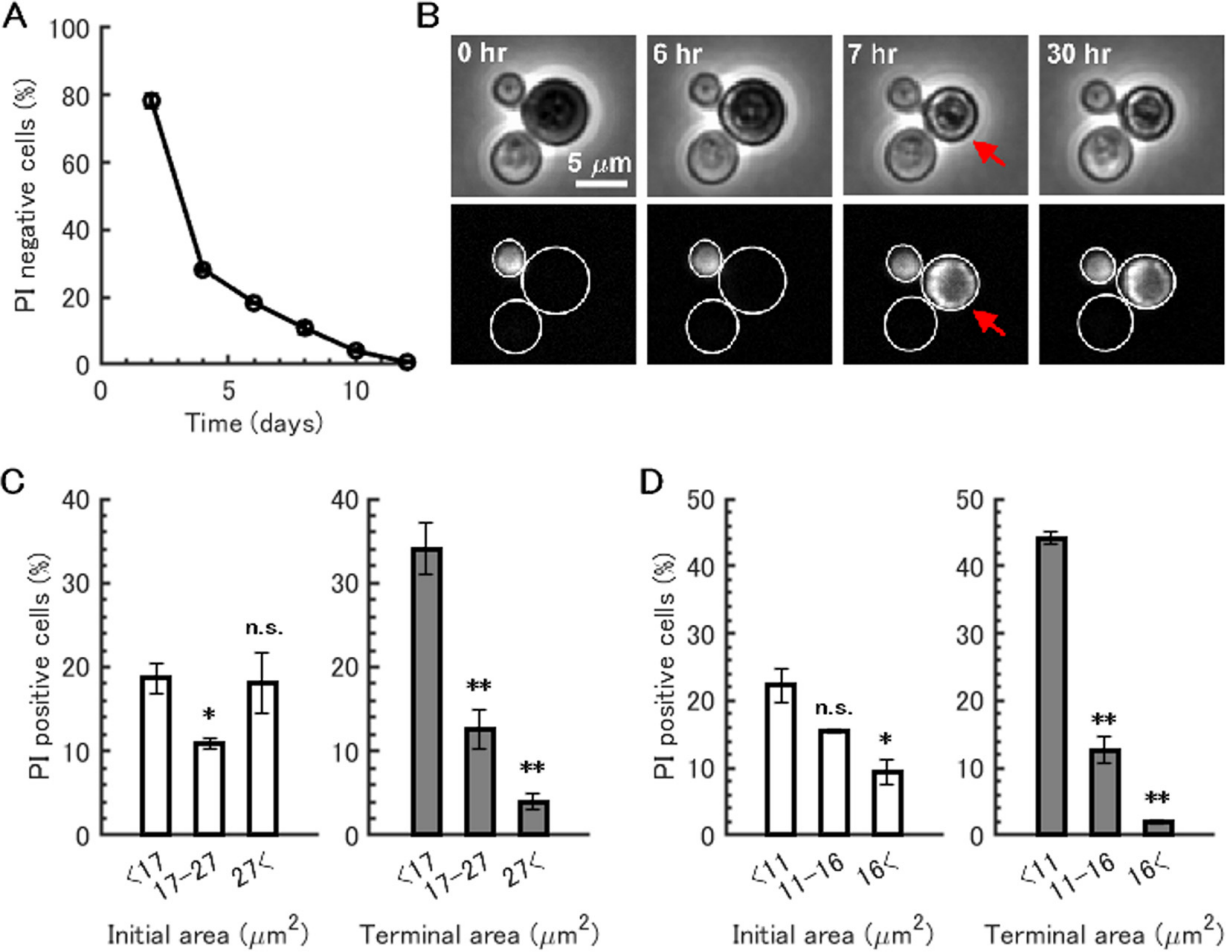
Size dependence of death rate in W303 and BY4741 strains. (A) Measurement of chronological life span during batch culture of S. cerevisiae W303 strain. Cell survival was estimated by the fraction of PI-negative cells. Mean survival from two independent experiments is shown. Error bars show standard errors of the means (SEM). (B) Time-lapse imaging of dying cells shows cell shrinkage that coincides with propidium iodide (PI) staining. Cells of W303 strain were cultured in SDC medium for 2 days and then placed on agarose pads containing conditioned medium and PI. Phase-contrast and fluorescence mCherry channel images are shown. A representative cell, which shrank in parallel with PI staining (*t* = 7 h), is indicated with a red arrow. (C) Death rate of cells with or without cell shrinkage effects in W303 strain stained with PI. Death rate was calculated against cell size at the beginning of the time-lapse period, i.e., excluding cell shrinkage effects (left, initial area), and against cell size at the end of the period, i.e., including cell shrinkage effects (right, terminal area). The size bin was set at the 33 and 66 percentiles of the distribution of initial cell area. The means (bars) ± SEM (error bars) for three independent experiments are shown. Two-sample *t* test was performed against the smallest size bin (n.s., not significant; *, *P* value < 0.05; **, *P* value < 0.01). (D) Death rate of cells with or without cell shrinkage effects in BY4741 strain stained with PI. Bar graphs were generated as described above for panel C.

Saccharomyces cerevisiae is often used as a model system to study aging and longevity. Longevity in this organism is defined in two ways—replicative life span (RLS) and chronological life span (CLS) (1). RLS is defined as the total number of daughter cells produced from a mother cell, and CLS is the period for which yeast cells can survive in the stationary phase (2, 3). It is well-known that the timing of cell death during chronological aging is not synchronized in a clonal population, suggesting that cell fate varies among individual cells (Fig. 1A). However, the cause of this heterogeneity is still unknown. Heterogeneity also exists in cell size in a clonal population. Previous single-cell observations found that mother cells of budding yeast increase in size during replicative aging (4, 5). From those observations, one may expect that larger cells die earlier than others due to aging in the stationary phase. Contrary to the expectation, several studies suggested that it might not be the case. Svenkrtova et al. observed that a subfraction of small cells, collected by centrifugal elutriation, showed a higher death rate than larger cells (6). Laporte and coinvestigators reported that replicative age, cell volume, and density did not affect the propensity to enter quiescence, while they observed more senescent cells (i.e., cells with globular mitochondria) in a fraction of small-sized cells (7). However, these past reports did not exclude a possibility that cells change their size in the process of or after cell death. If such a change exists, smaller or larger dead cells accumulate, leading to misinterpretations of size dependence of cell death. In this study, therefore, we performed time-lapse imaging of single cells in the stationary phase to revisit and ask whether the cell size correlates with the cell fate to die in chronological aging for better understanding of the cause of heterogeneity in cell survival.

This study used propidium iodide (PI) to distinguish dead and live cells in culture (Fig. 1A) (8–10). This method defined cell death as the loss of membrane integrity as described in reference 11. For time-lapse imaging, cells of S. cerevisiae W303 strain were grown in defined medium for 2 days. Cells were placed on agarose pads containing conditioned medium and PI. Cell size and its fluorescence were observed over 30 h at 1-h intervals. Out of 669 cells, 8.2% of cells were PI positive at the beginning of the time-lapse period. These cells were removed in the following analyses because we aimed to identify a property of cells that were about to die, not those already dead. During time-lapse observation, 15.1% of cells became PI positive (93 cells out of 614 cells) (Fig. 1B). To avoid the effects of any possible size changes upon or after cell death, we extracted the cell size information before cell death and compared death rates for groups of different cell sizes. Here, death rate was defined as the fraction of cells that became PI positive during time-lapse observation. When the death rate was calculated against cell size at the beginning of the time-lapse observation, no clear correlation between death rate and cell size was observed in the W303 strain (Fig. 1C, initial area). A similar analysis was also performed in the BY4741 strain, whose genetic background is different from that of strain W303. In this case, cells in the smallest size bin showed a higher death rate than cells in the biggest size bin (Fig. 1D, initial area). These data suggested that the dependency of death rate varied from one strain to another and that general principles do not exist. On the other hand, when the death rate was calculated against cell size at the end of time-lapse imaging, which could be affected by possible size changes due to cell death, the smallest size bin showed a significantly higher death rate than did the other size bins in both strains (Fig. 1C and D, terminal area). These results suggest that cells change their size upon or after cell death, and therefore, size before cell death must be considered when the relationship of cell size and cell fate is discussed.

**FIG 2:**
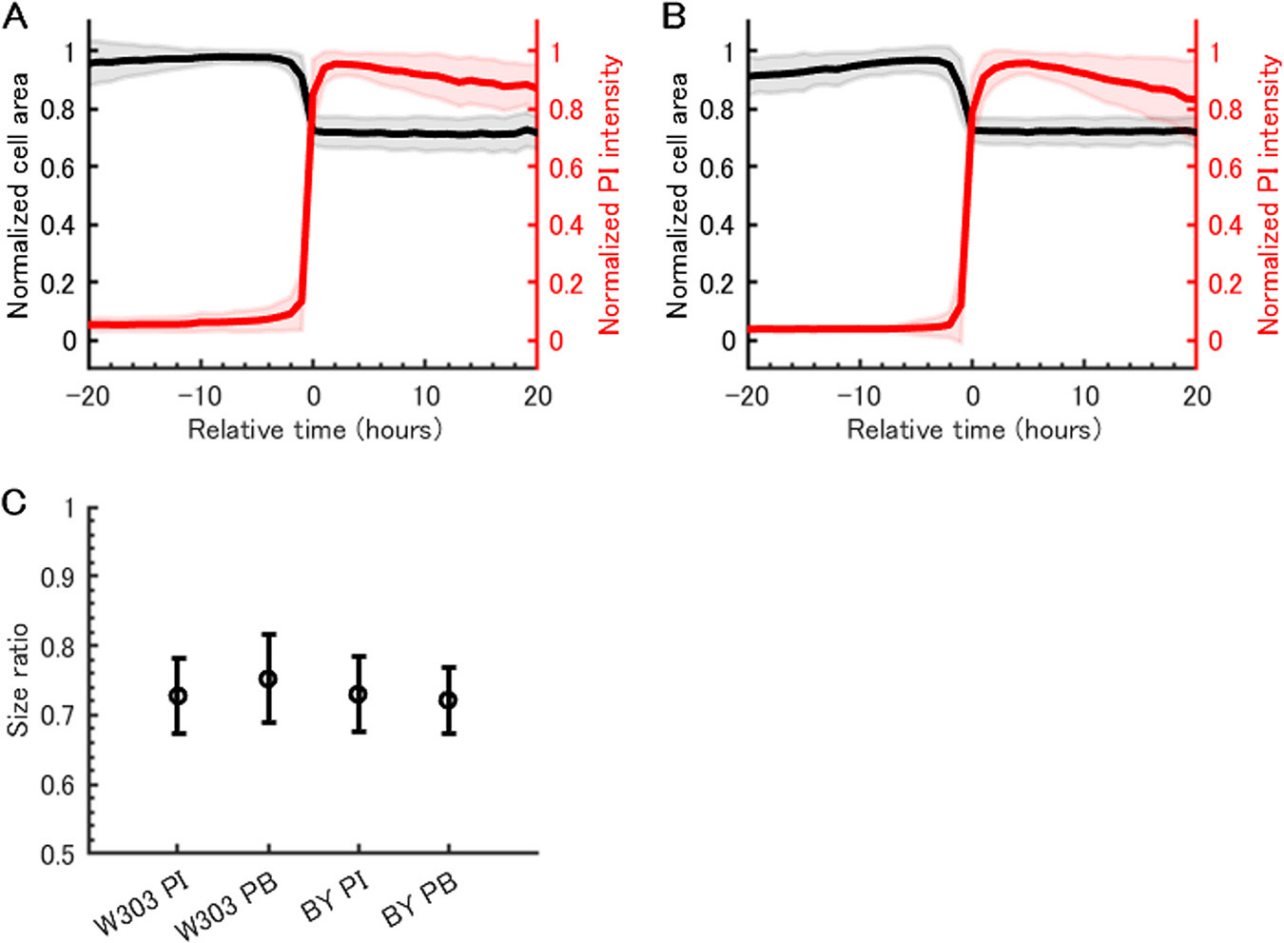
Cell shrinkage upon cell death. (A) Cell area and PI staining profiles in single cells of W303 strain over time. Cell area and PI intensity were normalized to the maximum value during the time-lapse. Cell area (black) and PI profiles (red) over time are shown in relative time, where time zero is defined when normalized PI intensity reaches 0.5. The lines show the mean for 93 cells from three independent experiments; shading shows the standard deviation (SD). (B) Cell area and PI staining profiles in single cells of BY4741 strain over time. The plot was generated as described above for panel A, with 262 cells from three independent experiments. (C) Degree of size reduction due to shrinkage upon cell death. The cell size ratio was calculated by dividing cell size after cell death by cell size before cell death. Four conditions were tested with propidium iodide (PI) or phloxine B (PB) staining in W303 and BY4741 (BY) strains. The mean size ratio for dead cells observed from three independent experiments is shown. Error bars depict SD.

From time-lapse observation, we noticed dying cells displayed a distinct phenotype from surviving cells (Fig. 1B). The dying cells shrank when they were stained with PI (Fig. 2A and B). The degree of shrinkage was proportional to the cell size before cell death, and it was 27.2% and 27.0% in the area in W303 and BY4741 strains, respectively (Fig. 2A, B, and C). The coefficient of variation (CV) was calculated as low as 0.075 in both strains. Less than 1% of surviving cells shrank more than 10% in the area during the time-lapse period, suggesting that the shrinking phenotype was specific to dying cells.

We wondered whether this shrinking phenotype is specific to the PI staining in this experimental setup, rather than indicating some biological features. To ask whether a similar phenomenon could be observed under different staining conditions, we used phloxine B, which is known to stain metabolically inactive cells (10, 11). We observed that cells shrank when they were stained with phloxine B, and the degrees of shrinkage were 24.8% and 27.9% in strain W303 and BY4741, respectively (Fig. 2C). Those data suggested that the shrinking phenotype is a common feature in the process of cell death. We also tested how culture conditions affected this phenotype. In complex medium, two phenomena were observed (see Movie S1 in the supplemental material). First, some cells were able to grow in the conditioned medium, resulting in several buddings. Second, dying cells seemed to burst rather than shrink, leaving small particles outside cells. More investigation will be needed to understand the dynamic process of cell death observed here and discuss the differences in cell behavior under these conditions.

10.1128/mBio.03094-21.1 MOVIE S1 Cells of W303 strain were grown in YPD medium for 15 days. Cells were placed on agarose pads containing the YPD conditioned medium and PI and incubated at 30°C. Time-lapse images were taken every hour. Combined images of phase-contrast and fluorescence microscopy in the mCherry channel are shown. Download Movie S1, AVI file, 0.5 MB. Copyright © 2021 Kato et al. 2021 Kato et al. https://creativecommons.org/licenses/by/4.0/ This content is distributed under the terms of the Creative Commons Attribution 4.0 International license.

In this report, we found that cell death was size dependent in a strain-specific manner. More importantly, we observed that cells shrank during the cell death process in chronologically aged cells grown in defined medium. Cell shrinkage was also observed when cells die after replicative aging (4, 12), suggesting different (chronological and replicative) aging processes undergo similar pathways when cells die. Therefore, cell shrinkage may be used as one of the reference points for cell death in this organism. An interesting observation is that the cell area shrinks 25 to 28% on average. It may indicate that cellular constituent(s) scaled with cell volume would cause cell shrinkage. Some organelles, such as the nuclei, vacuoles, and mitochondria, are scaled with cell volume in S. cerevisiae (13–15). Investigation of the contribution to the size reduction by loss or shrinkage of these organelles would be worthwhile. In the process of replicative aging, vacuoles were reported to be often ruptured at cell death (4). It is of interest whether vacuoles also play a role in cell death and shrinkage process in chronological aging. Our observation suggests that there are general principles and important mechanisms behind this shrinkage event for cell death. Identifying the cause and mechanism of the cell shrinkage will help us to understand the process of cell death and the origin of the heterogeneity in cell survival in a clonal population of S. cerevisiae.

## MATERIALS AND METHODS

### (i) Strain and culture condition.

The yeast strains used in this study were S. cerevisiae W303-1A (*MAT***a** *trp1-1 leu2-3 ade2-1 ura3-1 his3-11 can1-100*) and BY4741 (*MAT***a** *his3*Δ*1 leu2*Δ*0 met15*Δ*0 ura3*Δ*0*). Yeast cells were cultured at 30°C with shaking at 200 rpm in liquid SDC medium (0.17% yeast nitrogen base without amino acids and ammonium sulfate [Difco] but with 0.5% ammonium sulfate, 2% glucose, amino acids to a final concentration of 20 mg/liter for adenine, arginine, histidine, methionine, tryptophan, and uracil, 30 mg/liter for isoleucine, leucine, lysine, and tyrosine, 60 mg/liter for phenylalanine, and 150 mg/liter for valine) or YPD medium (1% yeast extract [BD], 2% Bacto peptone [BD], 2% glucose, 0.04% adenine, 0.02% uracil). The pH of SDC medium was adjusted to 6.0. For microscopic observation, cells were grown overnight in the indicated medium. These cells were diluted to 2 × 10^5^ cells/ml in fresh medium and incubated for the indicated times.

### (ii) Cell staining with PI.

Yeast cells grown in SDC medium were sampled at the times of interest and centrifuged at 5,000 rpm for 3 min (MX307; TOMY). After supernatants were discarded, cell pellets were suspended in phosphate-buffered saline (PBS) (0.8% sodium chloride, 0.144% sodium hydrogen phosphate, 0.02% potassium chloride, and 0.024% potassium dihydrogen phosphate). PI (Invitrogen) was added to the cell suspension at the final concentration of 0.33 μg/ml and incubated for 15 min at 30°C. After incubation, yeast cells were washed twice with PBS. Resuspended cells in PBS were used for microscopic observations. Cells with fluorescence in the mCherry channel over a threshold were identified as PI-positive cells. Exponentially growing cells were stained with PI by the same method, and the 95 percentile of the PI fluorescence profile for exponentially growing cells was used as a threshold.

### (iii) Microscopic observation and cell detection.

Microscopic observations were performed using an inverted microscope (Eclipse Ti2-E; Nikon), equipped with a SOLA light engine (Lumencor), an ORCA-flash4.0 v3 camera (Hamamatsu), and an object lens (Plan Fluor 40×/0.75; Nikon) with 1.5× optivar. Cells were placed onto a 1% agarose pad containing medium unless otherwise noted. Phase-contrast and fluorescence images with an mCherry filter block (Nikon mCherry HQ; excitation [Ex], 570/40; emission [Em], 645/75) were collected. Automated cell detection was performed with ImageJ and custom MATLAB (MathWorks) scripts. Cells were not detected if their area was smaller than 4.7 μm^2^. After cells were identified, each cell shape was fitted to an ellipse with open-source MATLAB code (16). All of the cell outlines were verified by manual inspection. Before the fluorescence intensity from single cells was extracted, fluorescence images were processed with a built-in function of ImageJ, Subtract Background.

### (iv) Time-lapse imaging and data analysis.

Cells were grown for 2 days (W303) or 1 day (BY4741) to the stationary phase. Conditioned medium was prepared by filtering the stationary-phase culture with a membrane filter (Millex-LG 0.20-μm Low Binding Hydrophilic LCR). Agarose pads (1% agarose) containing conditioned medium and 0.33 μg/ml PI or 5 ng/ml phloxine B were used for time-lapse microscopy. Cells at the stationary phase were spotted onto the agarose pad and imaged over 30 h at 1-h intervals with fully automated stage settings. During the time-lapse period, cells were kept at room temperature (about 24°C). Cell detection was performed as described above. At the end of the time-lapse imaging, the fluorescence profile of PI/phloxine B-stained cells could be divided into two subpopulations. One population showed high fluorescent signals, while the other showed very low signals, which is supposed to be the profile of surviving cells. Therefore, the minimum between these two peaks was used as a threshold to identify PI/phloxine B-positive cells. The degree of cell shrinkage was calculated by dividing the cell area after cell death by cell area before cell death. Here, the timing of cell death was defined as the timing when normalized PI/phloxine B intensity reached 0.5 as shown in Fig. 2A and B. Cell area before cell death was extracted by finding maximum cell area within 3 h before cell death. Cell area after cell death was extracted by finding minimum cell area within 3 h after cell death. If the cell size information was missing due to misdetection of the cell outline in those time windows, the cell was removed from the analysis. If cells were defined to be dead in the first or last 3 h of the time-lapse observation, those cells were also removed from the analysis.
